# A qualitative evaluation of an operational research course for acute care trainees in Kigali, Rwanda

**DOI:** 10.11604/pamj.2021.40.21.29191

**Published:** 2021-09-08

**Authors:** Tiffany Wang, Shannon Barter, Marcel Durieux, Tabor Flickinger, Theogene Twagirumugabe, Paulin Banguti

**Affiliations:** 1 School of Medicine, University of Virginia, Charlottesville, Virginia, United States of America,; 2 Anesthesiology Department, University of Virginia, Charlottesville, Virginia, United States of America,; 3 Department of Internal Medicine, University of Virginia, Charlottesville, Virginia, United States of America,; 4 Anesthesiology Department, University of Rwanda, Kigali, Rwanda

**Keywords:** Education, operational research, trainees, anesthesiology, Rwanda, LMIC, qualitative research

## Abstract

**Introduction:**

the blended SORT-IT model uses a combination of online modules and teleconferences with local and international mentors to teach operational research. We modified SORT-IT to create the Acute Care Operational Research (ACOR) course directed to anesthesiology residents in Kigali, Rwanda. This course takes students from an initial research idea through submitting a paper for publication. Our viewpoint on entering this study was that ACOR participants would have adequate resources to complete the course, but be hampered by cultural unfamiliarity with the blended teaching approach.

**Methods:**

we conducted a qualitative analysis of the experiences of all those who participated in the ACOR course to understand obstacles and improve future course iterations. Six anesthesiology residents participated in the first iteration of the course, with 4 local mentors and 2 secondary mentors, one of whom was based at the University of Virginia, with a total of 12 participants. Semi-structured interviews were conducted with all participants and mentors, which were independently coded for topics by two reviewers.

**Results:**

there was a 50% publication rate for those enrolled in the course and an expected 100% acceptance rate for those who completed the course. Some reported benefits to the course included improved research knowledge, societal improvements, and knowledge exchange. Some reported obstacles to successful course completion included time limitations, background knowledge, and communication. Of note, only 4 out of 12 participants recognized cultural barriers.

**Conclusion:**

although successful in the sense that all participants completed their research project, ACOR did not fully solve the main issues hindering research training. Our results show that research training in low-resource settings needs a continuing and formal focus on the factors that hinder participants´ success: mentorship and time.

## Introduction

The 1990 Commission on Health Research for Development, an international initiative, identified increased health research capacity as “one of the most powerful, cost-effective, and sustainable means of advancing health and development” in low- and middle-income countries (LMICs) [1, 2]. This statement sparked an increase in funding for projects to address research capacity in LMICs. Yet, the 10/90 report on health research showed that LMICs, representing 85% of the world´s population and 92% of the global disease burden, received only 10% of health research funding [3]. This “10/90” gap prompted a further expansion in resources dedicated to health research, frequently driven by high-income countries (HIC) with well-established health research infrastructure. Typically, this “vertical model” entails a HIC research collaborator traveling to a LMIC to implement a research project with minimal or short-term local involvement, returning to publish the work in their home country. Vertical health research relies on implicit capacity development, assuming that countries will build their research models following the examples of HIC partners. Projects dedicated to explicit capacity development take place in countries with pre-existing research infrastructure, thus creating “centers of excellence” critiqued as running parallel to the existing health system [1, 4].

Foreign-run research in LMICs has severely limited capacity development and research sovereignty of local systems. This is evidenced by the remarkable lack of published papers per capita in countries with low national gross product and total health expenditures, specifically African and former Soviet Union countries. In a 2012 study, six countries published fewer than three articles per 100,000 population, one of which was Rwanda [4].

Rwanda is a country with a promising future in health research capacity development. After the 1994 genocide, the Rwandan health system had a disastrous shortage of qualified healthcare providers. In addition to large-scale training of individuals in clinical practice, the country is actively increasing health research capacity, establishing a National Center for Clinical Research in 2011 [5, 6].

Operational research is used specifically to study how interventions with previously demonstrated efficacy can be applied in challenging settings, limited by high disease burden and resource scarcity [7]. The Structured Operational Research and Training Initiative (SORT-IT) empowers local infrastructure and systems in research capacity development. Overall, the self-paced research course seeks to address the lack of mentorship, time, and scientific writing skills that result in course failure, not leading to published papers. The course teaches protocol development, quality-assured data entry and analysis, and scientific paper writing [8].

The classic SORT-IT model is highly competitive, primarily focused on infectious disease research, and requires participants to travel to HIC countries for in-person instruction with their mentors for three separate weeks of modules, returning to their own countries in between to conduct research. This limits access to only those who can take time from their schedules and pay for travel and accommodation. A blended SORT-IT model was created to bypass these obstacles and increase accessibility. Teaching modules are offered online while students work individually or in groups, using a combination of email, teleconferences, and in-person meetings with local and international mentors [7]. The Acute Care Operational Research (ACOR) course was created based on this blended SORT-IT model, but focused on trainees in acute care medical specialties: anesthesiology, emergency medicine, surgery, and obstetrics. It is run over a full year, taking participants from an initial research idea to a submitted paper. The first iteration of the course was done in Rwanda with anesthesiology trainees due to the long-term, well-established relationship with this program.

Understanding obstacles and cultural issues faced by participants in this course is important to improve subsequent iterations of the program and aid implementation at other sites. We, therefore, conducted a qualitative analysis of participants´ experiences. We believed upon entering the study that ACOR participants would have adequate resources to complete the course, but be hampered by cultural unfamiliarity with the blended teaching approach.

## Methods

**Course structure:** the course, consisting of 3 modules, each a month in duration, was divided into units of video or software-based learning for students to complete on their own schedule; 5-10 hours per week study time was anticipated. Students were anesthesiology residents at the University Teaching Hospital of Rwanda (CHUK), and were asked to submit their work to a primary mentor weekly for revisions and commentary; the work was then sent to a more senior secondary mentor, who would evaluate the work for quality and timeliness. Senior residents at CHUK with previous research experience served as primary mentors, and were provided with the same modules and course materials as the students participating in the course. Secondary mentors were attending faculty at CHUK and the University of Virginia (UVA) with extensive research and teaching experience. Each week, students, primary and secondary mentors attended a teleconference for content presentations, discussions, and questions. Specific milestones were set for work completed during each module.

**Figure 1:**
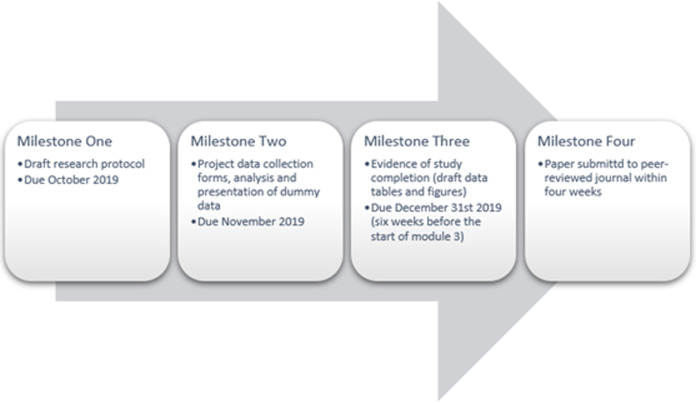
milestones and timeline

The first module consisted of video-based instruction on formulating research questions, writing a protocol, and preparing for ethics review. The milestone following this module was the completion of a draft research protocol. The second module consisted of tutorial-guided teaching to build a data collection tool able to provide input validation and analysis, all specifically using the EpiInfo^™^software package. The second milestone was completion of these data collection forms, with analysis and presentation of dummy data. Students were then given a 6-month period for data collection, analysis, and graph or table preparation in between the second and third modules. Throughout this time, there was continuous involvement of the primary mentors. As a part of a pre-existing research assistant program, students from the University of Virginia assisted in data collection and management due to their scheduled availability. At the end of this 6-month block, the SORT-IT students were expected to provide proof of study completion including data and figures. Module three focused on the writing component, and consisted of video-based instruction with secondary mentors on-site for about one to two weeks to provide writing feedback. The final product and milestone of this module was a paper submitted to a peer-reviewed journal within four weeks (Figure 1).

**Participants:** initially, 13 participants took part in the first iteration of the course: with 7 anesthesiology residents at the University of Rwanda, 4 local primary mentors, and 1 local and 1 international secondary mentor. One primary mentor dropped out of the program part-way through and one student filled his place, leading to a final count of 6 students, 4 primary mentors, and 2 secondary mentors.

After institutional review board approval was obtained, semi-structured interviews were conducted with all students and mentors in person, with the exception of one mentor, who was interviewed via Skype. Interviews were conducted prior to the end of the course but after data collection had been completed. All interviews were recorded and transcribed. A code book of themes that emerged in the interviews was iteratively developed by two reviewers until thematic saturation. All transcripts were independently coded using Dedoose^®^software, and any discrepancies between the coding of transcripts were reviewed and resolved collaboratively by both coders. Neither the interviewer nor the coders were otherwise associated with the course.

**Interview-guided course evaluation:** after the course was completed, semi-structured interviews were conducted with each course participant. They were asked what facilitated their completion of the course, what obstacles they faced, and what suggestions they offered for the next iteration of the course. Particular attention was paid to potential cultural obstacles. Separate questions were drafted for both the residents involved and their primary and secondary mentors. Interviews were conducted in person, except one that was done by phone, prior to the end of the course, but after data collection had been completed. The interviews were audio recorded and transcribed. The interviews were independently coded for topics by two reviewers. Discrepancies were resolved collaboratively by both reviewers, and a code book was iteratively developed.

## Results

Of the 6 participating students, 3 were able to complete and submit papers to the secondary mentors on time. Of the remaining 3, one struggled with a corrupted data file, which significantly delayed his progress; one had provided a draft paper by the time this manuscript was prepared, and one had not provided any written document. The three students who submitted their papers to their mentors were at various stages of publishing their work at the time of analysis (Table 1). As a result, there was a 50% publication rate for those enrolled in the course and an expected 100% acceptance rate for those who completed the course.

**Table 1:** course outcomes

Participant	Topic	Milestone 1	Milestone 2	Milestone 3	Milestone 4	Publication status by 01/2021
Student 1	ICU infection control	Complete	Complete	Complete	Complete	Published
Student 2	Critically ill obstetrics patients	Complete	Complete	Complete	Complete	Accepted
Student 3	Post-operative nausea and vomiting	Complete	Complete	Complete	Complete	In review
Student 4	Trauma pain management	Complete	Complete	Incomplete	Incomplete	Not published
Student 5	Chronic pain after hysterectomy	Complete	Incomplete	Incomplete	Incomplete	Not published
Student 6	ICU pain assessment and management	Complete	Complete	Complete	Incomplete	Not published

ICU: intensive care unit

All respondents said that the mentorship system and organization of the course were beneficial to the success of the course, in particular the use of milestones to guide students´ learning processes. Most also said that the international support was helpful in assisting the completion of the milestones. All the students found the course useful and said that it had improved their research knowledge. One student remarked: *“if I compare myself to my colleague who didn´t do the course, he still struggles in knowing these skills that I got from this course”*.

Others acknowledged that being able to apply skills learned in the course to an active project hastened their ability to utilize their knowledge and complete the project. One student participant noted that before the ACOR course: *“we didn´t know much about research, even writing a research protocol was difficult for us. But now we are comfortable doing - like the research protocol, during like one day you can do a research protocol”*. Additionally, a majority felt that the course had the potential to promote health in the country through a dedication to systems improvement with one primary mentor commenting: *“we have opportunity to convince donors, to convince government, to invest in healthcare, and investing in healthcare, it´s not only hospital, it´s investing in health professionals”*. Many participants discussed the benefits of exchanging knowledge with international participants and mentors in helping them develop research capability. Half of participants discussed publication as a benefit of enrollment in the course (Table 2). A student: *“I think from the ACOR course, a lot of people are going to publish, because it´s a channel through which we can go so that we can publish a lot of articles. Yes, it´s very important for us as researchers”*. A primary mentor also noted that the course: *“equips my department with strong research team mentorship which helps us publishing a lot, and also creating a better department where people can get their promotions and publish publications and residents don´t struggle to do their own studies which are not publishing"*.

**Table 2:** reported benefits of the course

Category	Description	Frequency	Percent of total
Improved research knowledge	Participant talks about the course having given them improved knowledge of how to conduct research or mentions it as motivation to continue	12	100%
Societal improvements	Participant mentions societal improvements as a result of course, such as increased quality of care, or otherwise helping their country/addressing problems	9	75%
Knowledge exchange	Participant mentions knowledge exchange as benefit of course or reason to continue	7	58%
Publication	Participant mentions publication as benefit of course or reason for interest	6	50%
Career opportunities	Participant mentions boost in career, attending conferences, etc. as benefits of the course that they see	3	35%
Network	Participant refers to gaining knowledge of how to work with a network of people not in the area, having met many people, etc	2	17%
Continuing current research project	Participant mentions continuing their current research project as a motive for interest in course	1	8%

Regarding obstacles, as documented in Table 3, all 12 interviewees said that limited time available to complete the course requirements was an issue. Both mentors and students struggled with time. Mentors commented on the difficulty facilitating the students´ paths through the course when they themselves had not reviewed the course modules given their workload outside the course. They struggled to find time to independently review the modules to a level of understanding where they could effectively help the students. A primary mentor commented: *“for the project mentors, most of them were on a volunteer basis, so the protected time was not easy to apply, so people were doing it on top of their job, which made it quite sometimes difficult to achieve the requirement”*. As a result of this lack of time, a secondary mentor noted that *“the quality of comments - for some of the comments, the quality of the comments was not at the level of a mentor”*.

**Table 3:** reported obstacles

Category	Description	Frequency	Percent of Total
Time	Participant mentions time as an issue	12	100%
Background knowledge	Themes relating to issues with background knowledge or lack thereof	11	92%
Communication	Themes relating to communication	11	92%
Infrastructure	Participant mentions issues with infrastructure that were an obstacle to the course (for example, internet access)	10	83%
Language	Participant mentions issues involving language	9	75%
Lack of face-to-face interaction	Participant discusses issues that arose from the reliance on videos/online communication. Separate from issues that arose due to distance from mentors	6	50%
Distance between mentor and student	Participant mentions issues regarding location of student or mentor in different city	5	42%
Cultural conflicts	Issues relating to cultural obstacles to the course such as importance of hierarchy	4	33%
Funding	Issues relating to funding	3	25%
Mentorship by people responsible for grading	Participant mentions themes relating to the use of mentors who are also responsible for the grades that the students receive in their residency	2	17%
Meeting attendance	Participant mentions issues around meeting or teleconference attendance, etc.	1	8%
Participant value	Participant has complaints about how valued they felt	1	8%
Technological issues	Problems related to technology, for example, software incompatibility	1	8%

Aside from limited mentor guidance, most students indicated that their extant workload and lack of protected time was an obstacle. One student pointed out the difficulty in completing their expected clinical duties with an assignment due weekly, saying: *“I was supposed to do all the duty of residents as required, and then add on this course, and it was difficult, and remember that each Sunday you have to submit some kind of milestone”*. In regard to balancing the course with other responsibilities, a participant expressed that: *“You have to be clinically good, you have duties you have to share with others, schedules...”*. Also pertaining to deadlines on particular sections of the course, a secondary mentor noted: *“there were some students - because of the deadline, who could submit half-cooked materials”*, suggesting that deadlines be extended. A student expressed: *“if you don´t have time, you cannot maximize all modules at the extent you want”*. Some students provided suggestions related to the deadlines: *“maybe submitting in two weeks, or kind of combining sections if possible”* or another participant suggested that deadlines be moved to Monday so that the entirety of the weekend was available for them to work on course materials. Several participants also discussed difficulties with efficient time use, with one mentor requesting of students that *“if the deadline is Sunday to submit work, people should start meeting ahead of time, like Monday, or do you see this new material, how you are working on it, can we met Wednesday to discuss on this. So, it helps working toward the timeline, toward the deadline, smoothly”*.

A majority said that a lack of background knowledge became problematic. Basic statistics and data visualization were specific areas of background knowledge that many felt they lacked, which negatively impacted their ability to succeed. One student noted: *“they ask you, for example, what kind of table to use, you don´t even know what kind of tables people do. I think someone needs to give you some guidance”*. A lack of sufficient research experience was also noted in the primary mentors with one of the secondary mentors pointing out that *“the methodology or scientific approach was a bit lacking for some”*. And one student mentioned how difficult it was to receive feedback from mentors, *“especially when you are starting this research thing that he does not know”*.

Most felt that limited communication between mentors and students was an obstacle, feeling that communication was infrequent, not rapid enough, or sometimes not helpful, and there were not enough primary mentors to satisfy their need for feedback. A student expressed: *“it was difficult for us to get like really really any input of mentors. It seemed like you were working alone”*. This prompted multiple comments on the need to escalate assistance to the secondary mentors, *“because he could help you immediately”*. Many felt that poor local infrastructure such as inconsistent access to Wi-Fi or functional computers slowed completion of the course. Some participants stated that language was an issue, with most indicating that difficulties with English were an obstacle in understanding the video module. Some also noted the challenges of language barriers when student data collectors arrived from the University of Virginia to help with data collection for their projects.

Of the twelve participants, only four people commented on cultural obstacles to the success of the course (Table 3). Two participants discussed a culture of strict hierarchy being an obstacle in the course, with one student commenting: *“because of our culture yeah, it´s about asking the boss. If you need the time and you try to get a respectful way to ask then he is not giving the positive answer, there is no way of keeping asking time, time, time. You stop there and you have to do what you have to do”*. Among the other two participants who discussed cultural conflicts, one participant mentioned a lack of time urgency, or an appreciation of an impending deadline associated with requests. Additionally, another participant mentioned a preference for face-to-face communication over email communication. However, the majority of people denied any cultural obstacles to their completion of the course.

## Discussion

This qualitative analysis of the participant-based interviews provides some insight into the possible reasons for our mixed success rate. Of the students who completed all the milestones in the ACOR course, we expect a 100% publication rate. Unfortunately, only 50% of the participating students were able to complete all the milestones at the time of writing, which indicates room for improvement. Studies that reviewed the SORT-IT course found a successful publication rate upwards of 90% [9-11]. When citing possible reasons for failure to complete the course, other authors found issues with ethics review, failure to collect or analyze data, or change in professional or social circumstances [10]. Little information is available on the elements of the course that possibly contribute to student failure or how the course could be improved, which we sought to amend with this study.

The students in this course generally found the mentorship system of the course and the stepwise progression of the course useful, but as a whole they found that the time they were given was insufficient. Time required to complete projects is a commonly cited issue in the building of health research capacity [1, 11]. Some SORT-IT reviews acknowledged that they specifically enrolled students into the course with a higher level of baseline experience in database management and descriptive statistics to compensate for the limited time, mentors available, and detailed lectures provided [7]. Some of the participants interviewed here highlighted the importance of needing to be very clinically competent in order to balance the requirements of the course and their own residency responsibilities. Options to address this include limiting the number of participants, so that a smaller amount of clinical work has to be taken over by the remaining providers, and spreading the course over a longer period of time.

The above identified issues concerning time can be directly linked to a paucity of mentors and their respective time, experience, and training. The students in this course felt that mentors did not communicate either frequently enough or with enough detail to be as helpful as desired. One review looking at outcomes from SORT-IT courses found that only 20% of enrolled participants continued on to become mentors. This was attributed to many of the course students coming from public health programs that were busy and overworked, who struggled to get time away from their work to complete course requirements, similar to our students. Participants in these circumstances must be highly and independently motivated to mentor as much of the work is completely voluntary [11]. The mentors in our study felt the same time strains, endorsing that it was difficult to provide proper guidance on the course when they were relatively unfamiliar with its demands. Like the students, they suffered from lack of dedicated time. However, the majority indicated that they were interested in mentoring subsequent students in the program despite the time and workload that the course would place upon them. We attribute many of these issues to this being the first year of the course. The mentors in a way were in the most difficult situation: they had no familiarity with the course and only little with the content, yet were expected to guide the students. It is imperative for course success that primary mentors are exposed to and/or trained on the material prior to the students´ involvement in the course, and lack of this was a definite weakness of the first course iteration. On the next iteration, students who completed this course will be able to serve as mentors and be thoroughly familiar with both the course and the content. The lack of experienced primary mentors with sufficient availability to assist students remains an important barrier to the implementation of this type of course in similar settings.

Overall, cultural issues were not found to be significant in preventing course completion. Cultural issues acknowledged pertained to the hierarchical structure of an academic setting that was maintained within the course´s mentoring system. Participants reported feeling uncomfortable escalating their needs to secondary mentors as it could be interpreted as disrespectful to the primary mentors. Participants ultimately relied heavily on one of the secondary mentors, an international participant and thus possibly seen as an outsider to the hierarchy, making him more readily accessible without the need to bypass social norms. Our observations are likely a reflection of a high-power distance, or the degree to which an organization rewards unequal distribution of power such that there is more power at higher levels of an organization or society. When comparing the construct of power distance between sub-Saharan African countries and the United States, for example, we see Africa overall averages higher power distance scores than the median United States score. High power distance societies tend to positively endorse characteristics of status consciousness and stricter procedural behaviors [12]. In future years, this course needs to recognize the difference in power structure between different societies to empower students to utilize resources in a way that is most comfortable. However, this issue aside, the majority of participants denied any significant impact from cultural factors on their completion of the course.

Limitations to this study include the limited sample size given that the course and the evaluation was conducted with only one anesthesiology residency program in Kigali, Rwanda. It remains possible that other programs in other parts of the region will not experience the same benefits and barriers to completion as were seen within this study. Other limitations include language barriers as the interviews were conducted primarily in English, which although is one of the official languages of Rwanda, may not be the language most comfortably spoken by all participants.

## Conclusion

These findings will be used to inform the next iteration of this course, which will include many of the suggestions provided by the participants. Our findings also can be useful to others initiating similar research courses, and have broader implications for strategies to improve research capacity in other comparable settings. The blended SORT-IT model provides several benefits to our participants. It enhances their research skills and productivity, using online modules to overcome barriers of distance and bring students and mentors together virtually. Half the students succeeded in submitting a paper to a peer-reviewed journal; several others will likely still do so. Additionally, our evaluation highlighted the potential challenges of infrastructure to support virtual learning and the importance of meeting the needs of both students and mentors in prioritizing research. Research training in low-resource settings needs a continuing focus on the key factors that hinder participants in being successful: experienced mentors and time. Future course development and evaluations of training programs should include formal assessment of these factors and improve support for participants in achieving their goals.

### What is known about this topic

- Research capacity is limited in low and middle-income countries, indicating the need for an education infrastructure on conducting relevant health research projects;
- The structured operational research and training initiative has been successful in improving meaningful research output in these countries.

### What this study adds

- The acute care operational research course, a modification of the blended SORT-IT course, subjectively improved the research skills and productivity of anesthesiology residents in Kigali, Rwanda; this finding indicates that this course would be a useful model to improve research capacity in this country through a structured, semi-virtual training program in operational research;
- The limitations highlighted in this course evaluation are useful to improve future iterations of the course in Kigali and to establish effective research educations programs in other LMICs.
